# Different elevational patterns of rodent species richness between the southern and northern slopes of a mountain

**DOI:** 10.1038/s41598-017-09274-2

**Published:** 2017-08-18

**Authors:** Ling-Ying Shuai, Chun-Lei Ren, Wen-Bo Yan, Yan-Ling Song, Zhi-Gao Zeng

**Affiliations:** 1 Key Laboratory of Animal Ecology and Conservation Biology, Institute of Zoology, Chinese Academy of Sciences, Beijing, 100101 China; 2 College of Life Sciences, Huaibei Normal University, Huaibei, 235000 China; 3 Bio-resources Key Laboratory of Shannxi Province, Shannxi Sci-Tech University, Hanzhong, 723001 China

## Abstract

Studies on elevational gradients in biodiversity have accumulated in recent decades. However, few studies have compared the elevational patterns of diversity between the different slopes of a single mountain. We investigated the elevational distribution of rodent diversity (alpha and beta diversity) and its underlying mechanisms along the southern and northern slopes of Mt. Taibai, the highest mountain in the Qinling Mountains, China. The species richness of rodents on the two slopes showed distinct distribution patterns, with a monotonically decreasing pattern found along the southern slope and a hump-shaped elevational pattern evident along the northern slope. Multi-model inference suggested that temperature was an important explanatory factor for the richness pattern along the southern slope, and the mid-domain effect (MDE) was important in explaining the richness pattern along the northern slope. The two slopes also greatly differed in the elevational patterns of species turnover, with the southern slope demonstrating a U-shaped curve and the northern slope possessing a roughly hump-shaped pattern. Our results suggest that even within the same mountain, organisms inhabiting different slopes may possess distinct diversity patterns, and the underlying mechanisms may also differ. The potential role of the factors associated with slope aspect in shaping diversity, therefore, cannot be ignored.

## Introduction

Understanding biodiversity distribution patterns and the mechanisms behind them are fundamental and challenging tasks for ecologists. During recent decades, elevational patterns of biodiversity have received increasing attention^1, 2^. Mountains are important to ecologists not only because they occupy a large part of Earth’s land surface and often possess high biodiversity but also because they act as powerful natural experimental systems^3^. Compared to latitudinal gradients, mountains often encompass significant variance in many important ecological factors at a much smaller scale, which enables researchers to separate thermal factors from seasonal effects^4^. Reasonably, elevational gradients in biodiversity were once thought to be analogous to latitudinal gradients^5^. In this sense, diversity should decrease monotonically with increasing elevation, and this was once treated as a general pattern^5, 6^. However, a quantitative review suggested that only a small portion of empirical studies supported this idea^6^. In many cases, hump-shaped relationships were found, with the species richness peaking at mid-elevations^7–12^. In other cases, the richness curves seemed to peak in the foothills and plateau at high-elevations^13^. In other words, there appears to be no such a general pattern of elevational gradients in species richness.

Similarly, our understanding of the mechanisms behind the elevational patterns of diversity remains limited. However, some environmental factors seem to play important roles in generating such patterns. Among these driving factors, climate may be the most widely accepted. On the one hand, climate may act as a species filter because species are spatially limited by their physiological tolerances^14, 15^. On the other hand, gradients of some climatic factors (such as precipitation, temperature and range of temperature) generate gradients of energy availability and primary productivity, a basis for the generation and maintenance of diversity. In a highly cited review, Hawkins *et al*. concluded that in large-scale terrestrial studies, variables of water, energy and water-energy balance successfully explained most of the spatial variance in species richness across a wide range of flora and fauna types^16^. Climatic factors are thus viewed as important predictors of large-scale patterns of biodiversity^15–17^, and their roles in shaping species richness have been supported by many empirical studies^10, 12, 18, 19^. However, fine-scale climatic data are often hard to obtain, which greatly hinders the comprehensive assessment of the influence of climatic factors^20^.

Although environmental gradients are often important in generating gradients of diversity, the role of geometric factors cannot be ignored. For example, area is a well-documented geometric factor. It is widely accepted that a larger area should harbour more species because of higher habitat heterogeneity and lower extinction rates^21–23^. Just as the latitudinal decrease in diversity largely reflects the latitudinal decrease in land area^23^, the monotonically decreasing pattern found in elevational transects can sometimes be attributed to the elevational decline in land area. After being standardized for area, a formerly monotonically decreasing richness-elevation curve may then become hump-shaped^6^. Another important geometric factor is the mid-domain effect (MDE), which predicts that even without any impacts of ecological or evolutionary processes, species richness should tend to peak at a mid-elevational zone (rather than the previously thought uniform pattern). The MDE stems from the idea that the ranges of species randomly placed between hard boundaries (e.g., summits, rivers and seas) would increasingly overlap towards the midpoint of the domain^24, 25^. Generally, the MDE is more significant on larger-ranged species since these species are more spatially constrained^10, 19, 26, 27^. Although more or less controversial^28, 29^, the MDE can be used as a null model to test against^27^ and has recently been tested and verified in many studies^9, 10, 12, 19, 30–32^.

Although studies on elevational patterns of diversity have accumulated over the past several decades, surprisingly few studies have compared such patterns between different slopes of the same mountain. Due to the hampering effect of mountain bodies, the southern and northern slopes of the same mountain (especially those at mid latitudes) often receive quite different amounts of solar radiation and precipitation^33^. Accordingly, climate, soil and vegetation characteristics often differ between the southern and northern slopes^34–36^. Previous studies have also suggested that slope aspect significantly affects the habitat selection of many species^37–39^. It is reasonable to expect that even within a single mountain, the elevational patterns of diversity may also vary between the gradients with different slope aspects.

Unlike species richness, species turnover (or beta diversity) measures the difference in species composition between assemblages^40^. The concept of species turnover is important as it reflects habitat partitioning among species and enables us to compare habitat diversity between study systems^41^. Species turnover is also considered as a key driver of the global patterns of species richness^42^. Due to their dramatic changes in climate, vegetation and topography, mountains are ideal study systems to explore the patterns and the underlying mechanisms of species turnover^43^. However, while elevational patterns of species richness have been well documented, patterns of elevational species turnover have received limited attention^44^. Meanwhile, the relationship between species turnover and elevation remains controversial^44^.

**Figure 1:**
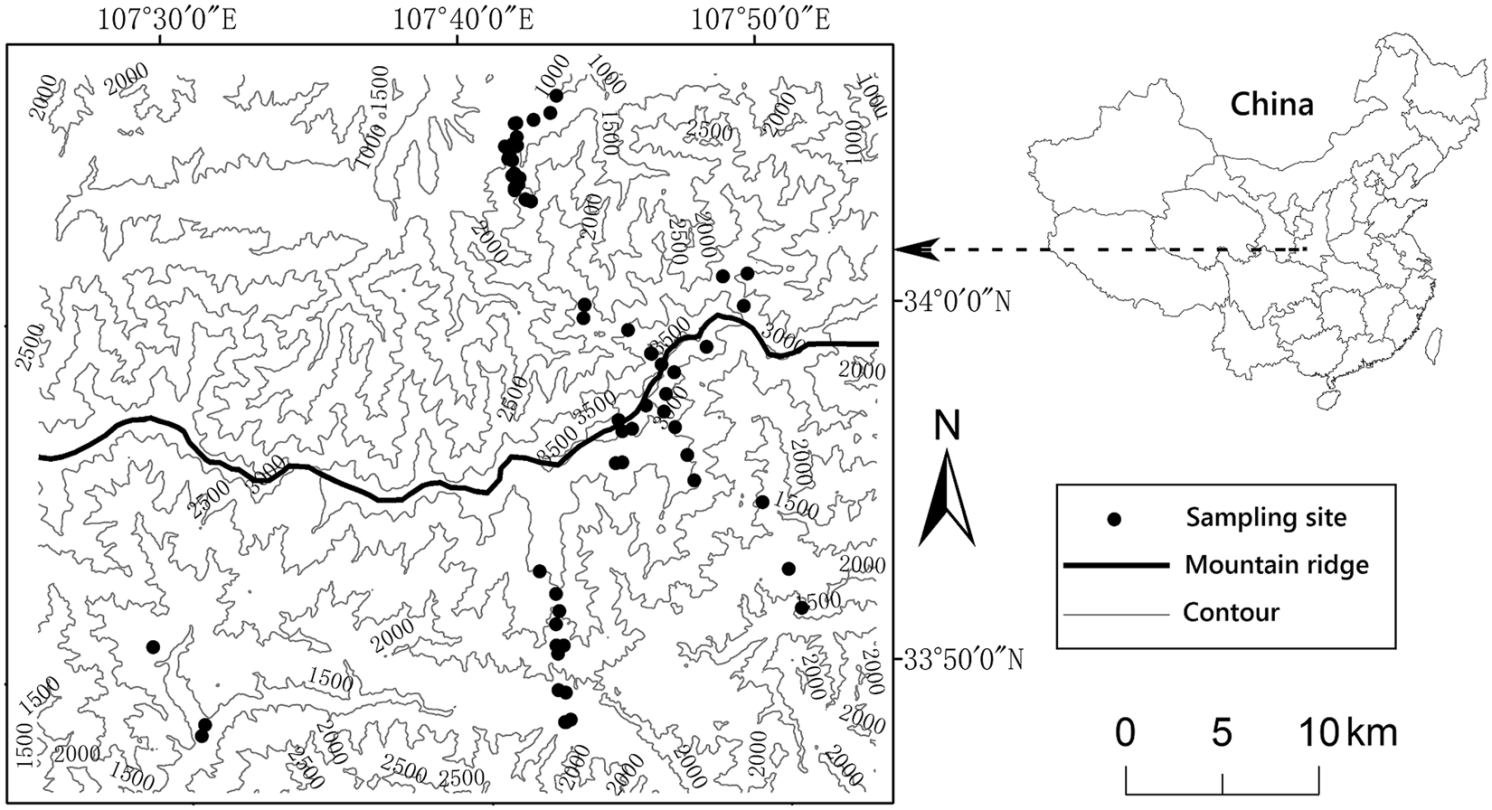
Map showing the study area and the sampling sites. The map was created with ArcGIS 10.1 (www.arcgis.com).

In this study, we seek to explore the patterns of elevational diversity in rodents on the southern and northern slopes of Mt. Taibai, the highest mountain (3767 m a.s.l.) in the Qinling Mountains, China. The Qinling Mountains form an ecotone between the subtropical and warm temperate zones and play important roles in the formation of the regional patterns of biodiversity in China. Mt. Taibai possesses a roughly west to east arrangement (Fig. 1), with the southern and northern slopes differing in vegetation and climate^35, 45^, which makes it suitable to compare the two slopes. We then evaluate the relative importance of five frequently mentioned explanatory variables (temperature, precipitation, primary productivity, area and the MDE) in forming richness patterns by conducting multiple regressions and multi-model inference. Previous studies have systematically measured the elevational patterns of some climatic factors on the southern and northern slopes of Mt. Taibai at a relatively fine scale^35, 45^. The results of these studies help us explore the roles of climatic drivers in shaping the patterns of diversity. Furthermore, we intend to assess several assumptions associated with species turnover. First, according to the null mid-domain models, species turnover along each gradient should decline towards the mid-elevation area, thus generating a U-shaped curve^42^. Second, according to the well-documented distance decay of similarity, the dissimilarity between paired assemblages should increase with geographic distance^46, 47^. Finally, since the mountain ridge is possibly the most important path across which animals disperse between the slopes, paired communities on different slopes with similar elevations would become less isolated from each other as the elevation increases. Under this scenario, we predict that the difference in local species composition between slopes would decrease with increasing elevation since dispersal has proved to be an important driving factor in generating the distance decay of similarity.

## Results

A total of 30 rodent species (pikas were also included because of their close relationship with rodents) were recorded, including Muridae (9 species), Cricetidae (6 species), Sciuridae (5 species), Petauristidae (3 species), Platacanthomyidae (2 species), Zapodidae (2 species), Rhizomyidae (1 species), Hystricidae (1 species) and Ochotonidae (1 species). Of these species, 18 belonged to the Oriental realm, and the other 12 belonged to the Palaearctic realm. A comprehensive list of the species and the elevational range for each species can be found in Supplementary Table S1.

**Figure 2:**
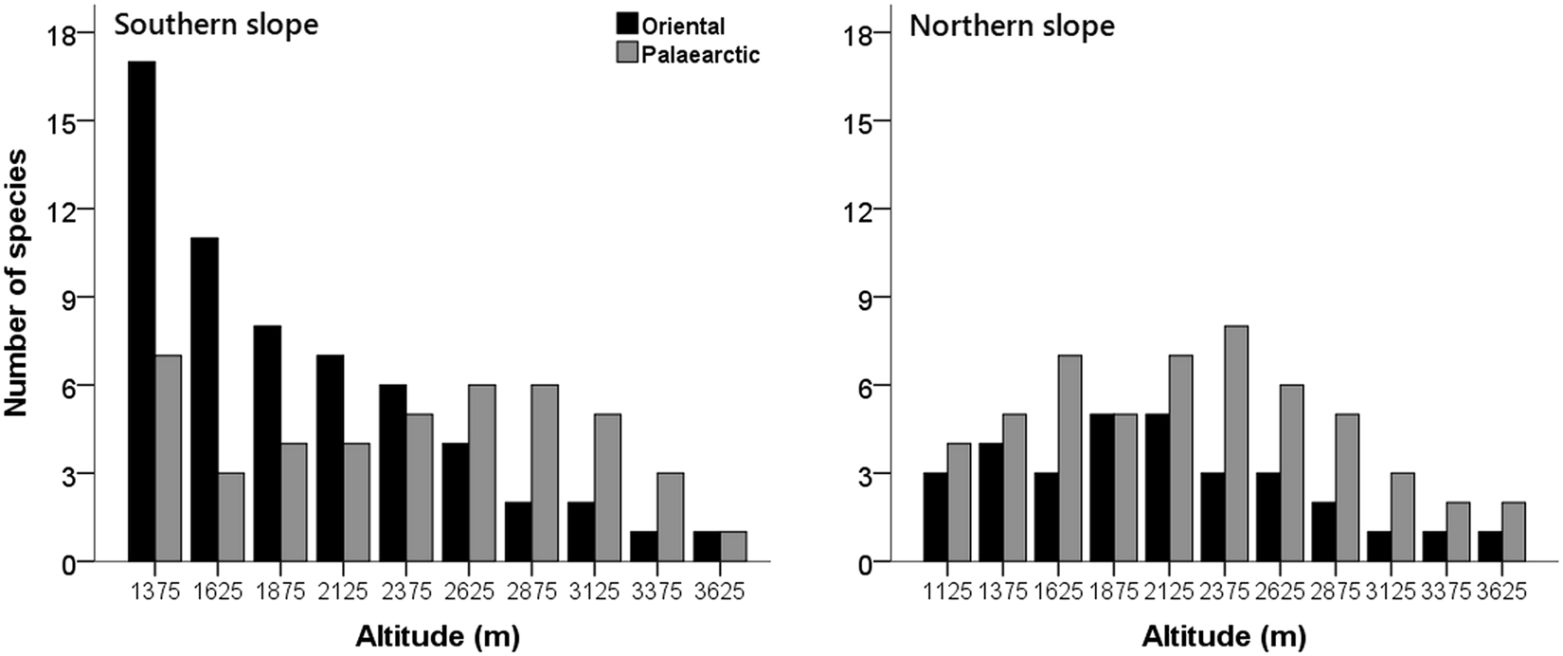
The elevational distribution of Oriental and Palaearctic rodent species (interpolated) along the southern and northern slopes of Mt. Taibai, China. The horizontal axis indicates the midpoints of the elevational bands.

The southern slope (29 rodent species) possessed more rodent species than the northern slope (18 rodent species). A total of 18 out of 29 species (approximately 62%) on the southern slope and 6 out of 18 species (approximately 33%) on the northern slope belonged to the Oriental realm. A total of 17 species were shared by both slopes, 7 of which were Oriental species. However, species of Petauristidae, Platacanthomyidae, Rhizomyidae and Hystricidae were only recorded on the southern slope, while *Myospalax smithi* was only found on the northern slope. With increasing elevation, the southern slope contained fewer Oriental species (Fig. 2). The distribution of Palaearctic species along the southern slope was relatively irregular, with two peaks occurring at approximately 1300 m a.s.l. and 2700 m a.s.l. (Fig. 2). The polynomial regressions suggested that both the Palaearctic and Oriental species along the northern slope followed hump-shaped elevational patterns (quadratic regression: Palaearctic, *r* ^2^ = 0.79, *P* = 0.002; Oriental, *r* ^2^ = 0.73, *P* = 0.006; Fig. 2).

**Figure 3:**
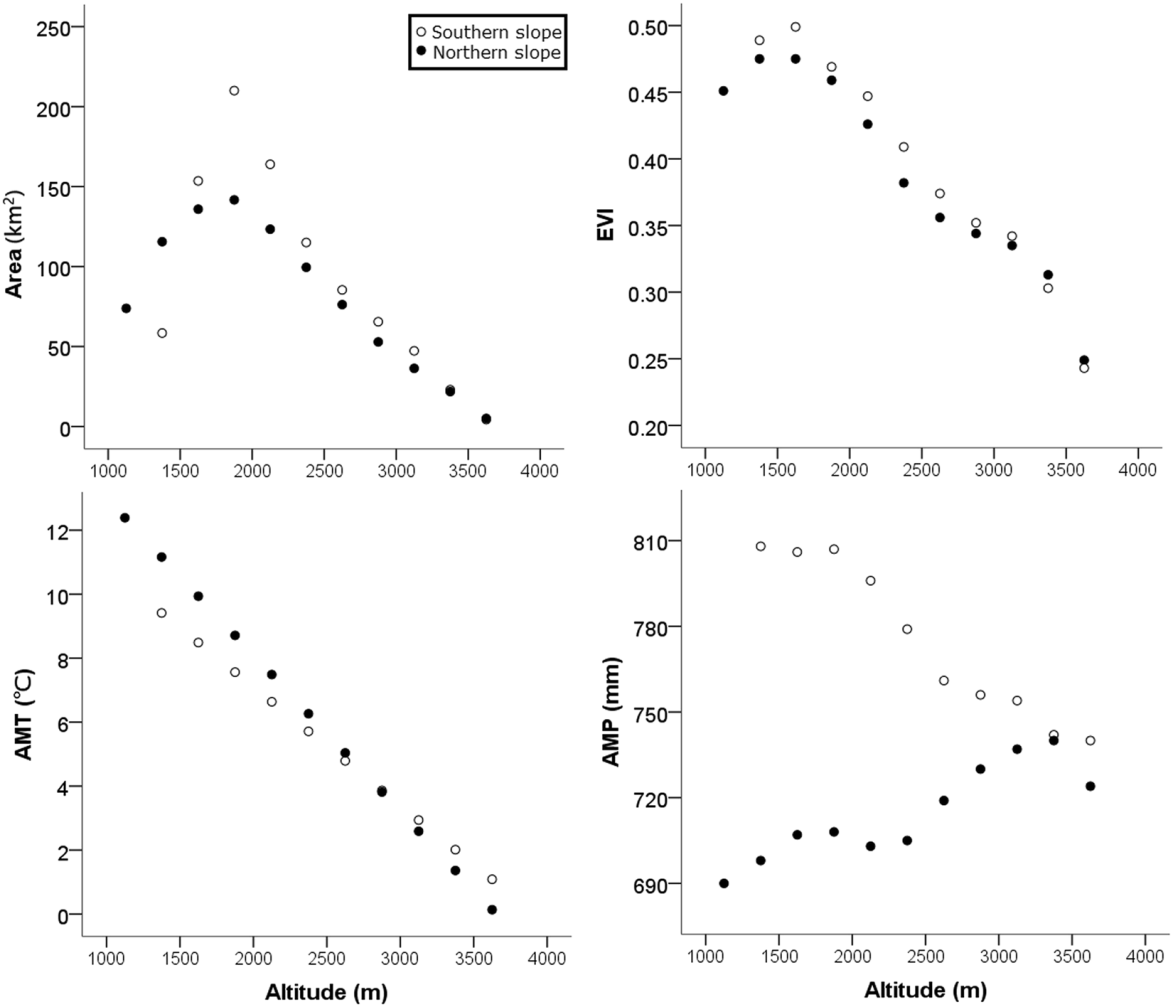
The elevational distribution of the environmental variables and surface area along the southern and northern slopes of Mt. Taibai, China. AMT: annual mean temperature; AMP: annual mean precipitation; EVI: enhanced vegetation index.

The two slopes of Mt. Taibai differed in some explanatory factors (Fig. 3). The southern slope had higher precipitation and primary production, as suggested by the annual mean precipitation (hereafter AMP; paired samples *t*-test: *t* = 4.42, *df* = 9, *P* = 0.002) and enhanced vegetation index (hereafter EVI; paired samples *t*-test: *t* = 2.93, *df* = 9, *P* = 0.02), respectively. Although one may think that the southern slope should be warmer, the two slopes did not differ significantly in terms of annual mean temperature (hereafter AMT; paired samples *t*-test: *t* = −1.92, *df* = 9, *P* = 0.09; Fig. 3). The two slopes also did not differ significantly in terms of area (paired samples *t*-test: *t* = 1.17, *df* = 9, *P* = 0.27). On both slopes, the area-altitude curves were hump-shaped rather than monotonically decreasing (Fig. 3).

**Figure 4:**
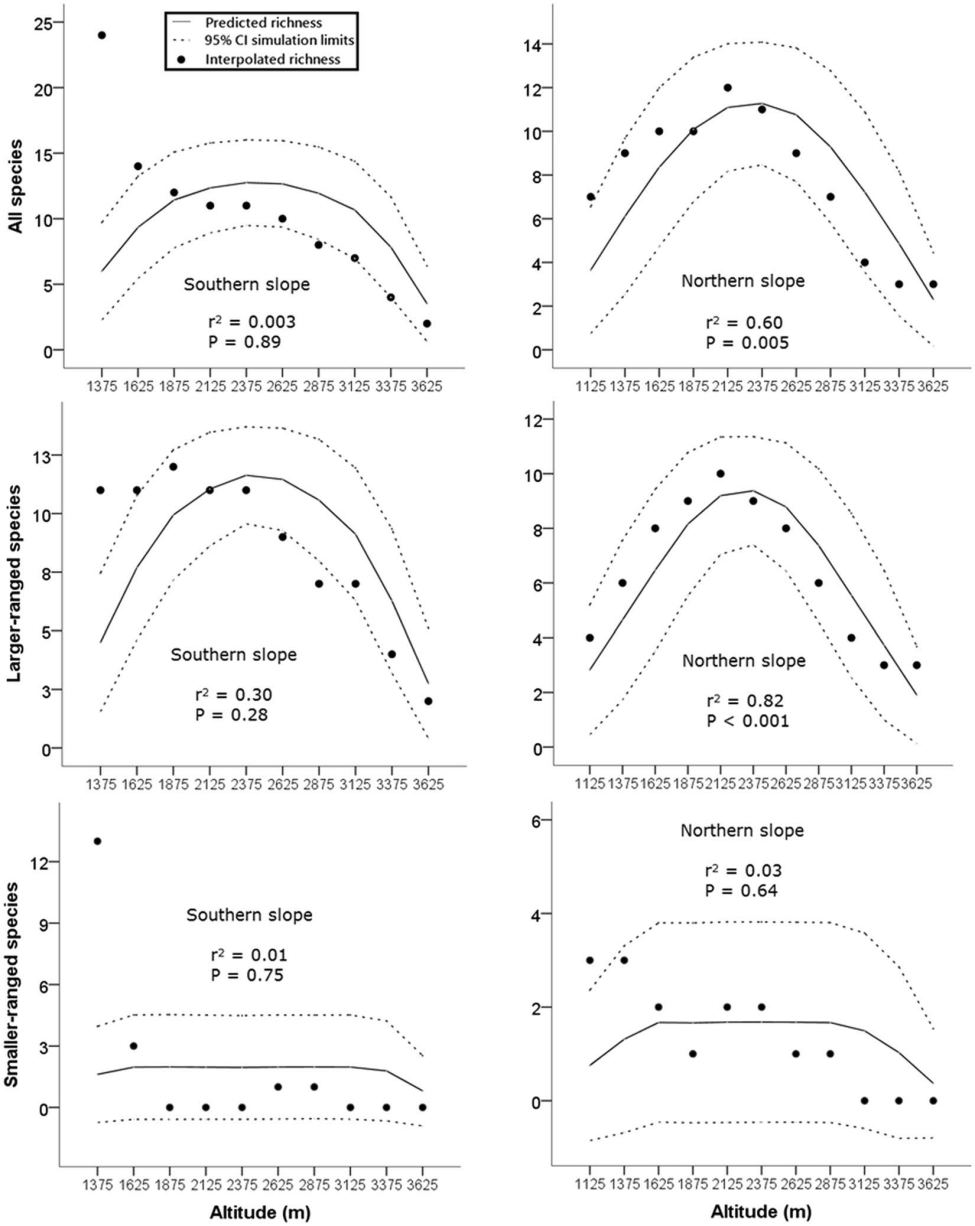
Rodent species richness (interpolated) along the elevational gradients on the southern and northern slopes of Mt. Taibai, China, with predicted richness and its 95% simulation limits of the mid-domain null models generated from 50,000 simulations. The *r* ^2^ and *P* values for each linear regression of the observed richness against the predicted richness are presented to indicate the fit of the null mid-domain effect models. Larger-ranged and smaller-ranged species are the species with elevational ranges above and below the median range size, respectively. The horizontal axis indicates the midpoints of the elevational bands.

In terms of rodent species richness, the southern and northern slopes of Mt. Taibai possessed distinct elevational patterns (Fig. 4). Although both gradients were best summarized by the third-order polynomial regression models according to the corrected Akaike Information Criterion (hereafter AIC_c_) values (Supplementary Table S2), the total species richness along the northern slope was hump-shaped with a peak occurring at approximately 2000 m a.s.l. The total species richness along the southern slope decreased with increasing elevation (Fig. 4). The elevational richness patterns of the larger-ranged species along both slopes were also hump-shaped, while those of the smaller-ranged species were irregular but generally decreased with increasing elevation (Fig. 4). The linear regressions suggested that when analysed separately, the MDE was a good explanatory factor for the richness patterns of all species (*r* ^2^ = 0.60, *P* = 0.005) and the larger-ranged species (*r* ^2^ = 0.82, *P* < 0.001) along the northern slope (Fig. 4). However, the explanatory power of the MDE for the distribution of species on the southern slope and the smaller-ranged species was weak (*P* > 0.05; Fig. 4).

The multiple ordinary least squares (hereafter OLS) regressions (without considering the effects of spatial autocorrelation) suggested that the richness patterns along the two gradients were driven by different factors (Table 1). As the *β* coefficients for the best-fit models suggested, the species richness along the northern slope was positively correlated with values predicted by the MDE null model, but negatively correlated with the AMP. The AMT was significantly correlated with the rodent species richness along the southern slope (Table 1). The cross-model validation suggested similar results, with the AMP (negative) and the MDE perceived as important (i.e., impact factor ≥ 0.80) along the northern slope, while the AMT was important along the southern slope (Table 2). For the larger-ranged species on the northern slope, both geometric factors (area and the MDE) were important in explaining the richness patterns according to the best model and the cross-model validation. For the larger-ranged species on the southern slope, the MDE and the AMT were important explanatory factors in the best model, and the MDE was also considered important by the cross-model validation. For the smaller-ranged species along the southern slope, the AMT was an important explanatory factor. The AMP was negatively correlated with the richness patterns of smaller-ranged species along the northern slope in the best OLS model. No factor was considered important for the smaller-ranged species along the northern slope in the cross-model validation (Table 2). In some of the best-fit models, area was negatively related to species richness, which suggested that the effect of area was overridden by the effects of other factors since it is assumed that a larger area harbours more species. For a summary of all 31 models, see Supplementary Tables S3–S8.

**Table 1:** Multiple ordinary least squares regressions on species richness against five explanatory factors for different species groups on the southern and northern slopes of Mt. Taibai, China. Only the results of the best model and the model averaging for each gradient are presented, and the model selection is based on the lowest Akaike Information Criterion (AIC_c_) values. Larger-ranged species: the 50% of species with ranges above the median range size; smaller-ranged species: the 50% of species with ranges below the median range size. MDE: mid-domain effect; AMT: annual mean temperature; AMP: annual mean precipitation; EVI: enhanced vegetation index. For a summary of all 31 models, see Supplementary Tables S3–S8.

	Southern slope						Northern slope					
	Best model			Model averaging			Best model			Model averaging		
	*β*	*S.E*.	*t*	*β*	*S.E*.	*t*	*β*	*S.E*.	*t*	*β*	*S.E*.	*t*
*Total species*												
Area	−0.50	0.01	−5.54	−0.52	0.01	−5.09				0.22	0.00	10.11
MDE				0.16	0.01	23.23	0.70	0.06	11.70	0.68	0.08	9.15
AMT	1.27	0.20	14.03	1.28	0.22	12.48				−0.04	0.01	−7.33
AMP				0.45	0.01	16.61	−0.62	0.01	−10.41	−0.60	0.02	−7.34
EVI				0.60	1.72	24.99				0.07	0.24	12.47
*Larger-ranged species*												
Area				−0.25	0.00	−15.53	0.93	0.01	7.07	0.89	0.01	3.92
MDE	0.39	0.07	6.67	0.40	0.08	5.23	0.49	0.06	8.24	0.51	0.09	5.62
AMT	0.84	0.07	14.25	0.82	0.07	15.20				−0.16	0.08	−1.25
AMP				0.84	0.01	15.58	−0.21	0.01	−3.72	−0.27	0.01	−3.75
EVI				0.79	0.78	40.94	−0.59	3.67	−5.49	−0.64	0.09	5.62
*Smaller-ranged species*												
Area	−1.08	0.01	−10.16	−1.10	0.01	−8.46				−0.11	0.00	−5.42
MDE				−0.10	0.03	−32.78				0.06	0.03	4.55
AMT	1.36	0.15	12.85	1.38	0.20	9.96				0.93	0.06	4.57
AMP				1.27	0.01	25.64	−0.92	0.01	−6.84	−0.78	0.01	−5.50
EVI				−0.49	1.59	−14.69				−0.28	1.56	−2.68

**Table 2:** Impact factors for each explanatory factor per gradient. The impact factors are the sum of the Akaike weights for each model including the explanatory factors across the whole model set. The bold values represent the factors with strong support (larger than 0.80) across all 31 models. Larger-ranged species: the 50% of species with ranges above the median range size; Smaller-ranged species: the 50% of species with ranges below the median range size; MDE: mid-domain effect; AMT: annual mean temperature; AMP: annual mean precipitation; EVI: enhanced vegetation index.

	Southern slope			Northern slope		
	*Total species*	*Larger-ranged species*	*Smaller-ranged species*	*Total species*	*Larger-ranged species*	*Smaller-ranged species*
Area	**0.99**	0.11	**1.00**	0.17	**0.98**	0.07
MDE	0.06	**0.99**	0.03	**1.00**	**1.00**	0.07
AMT	**0.98**	0.62	**0.92**	0.03	0.28	0.58
AMP	0.03	0.31	0.10	**1.00**	0.76	0.56
EVI	0.03	0.11	0.05	0.03	0.73	0.17

**Figure 5:**
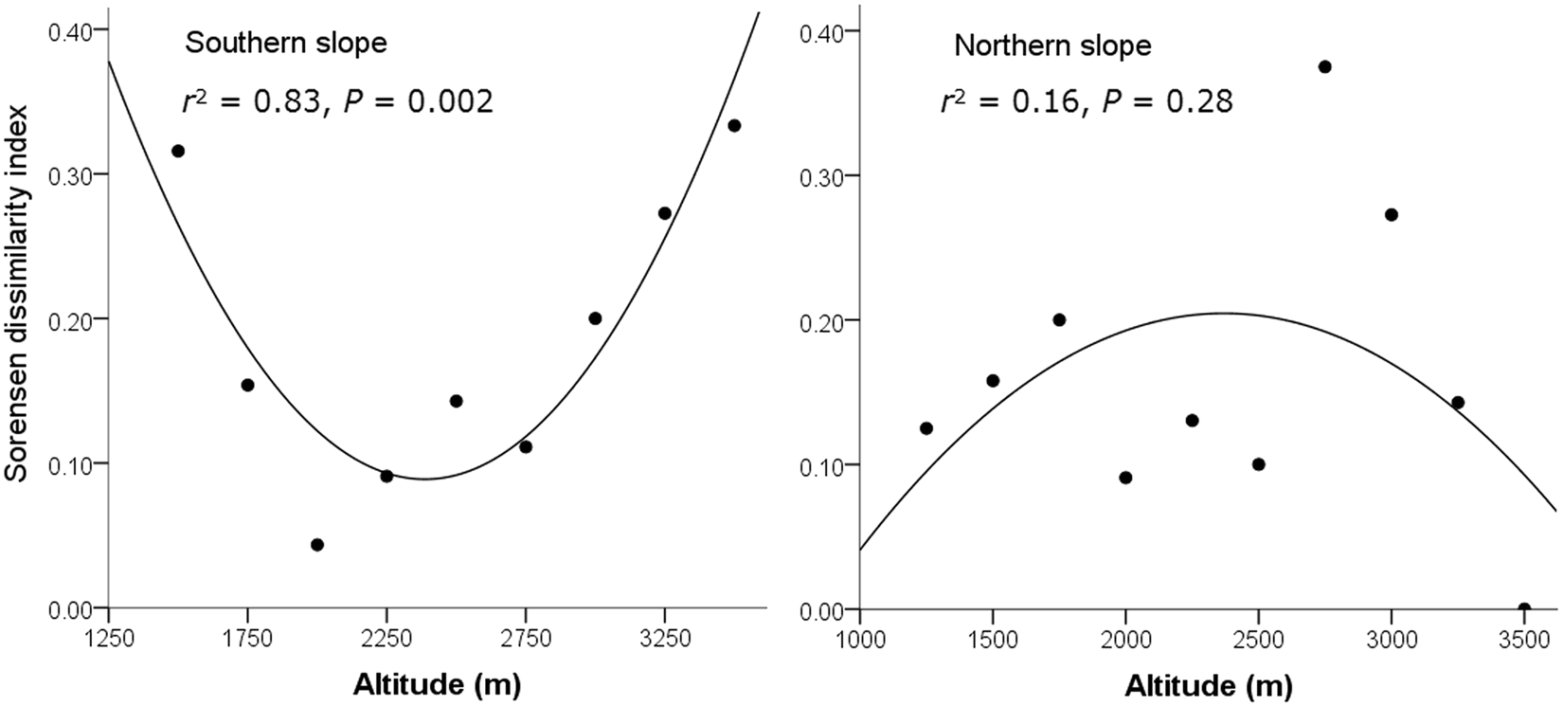
The elevational distribution of the Sorensen dissimilarity index along the southern and northern slopes of Mt. Taibai. The results of the polynomial regressions are presented.

**Figure 6:**
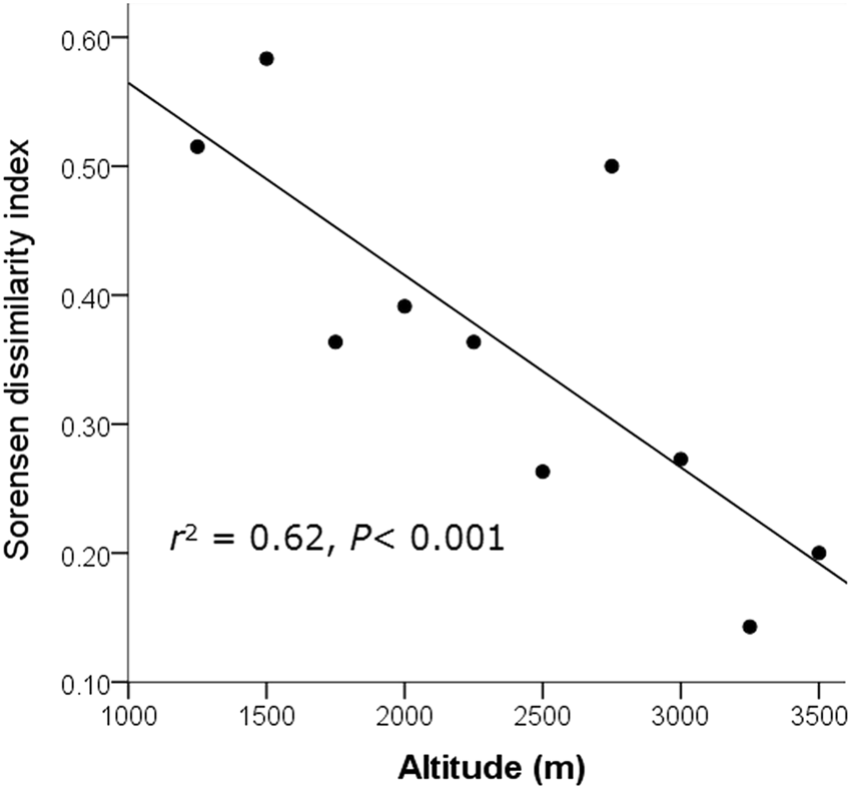
The elevational distribution of the Sorensen dissimilarity index between paired assemblages of similar elevation on the southern and northern slopes of Mt. Taibai. The results of the linear regression are presented.

The two slopes possessed quite different elevational patterns in beta diversity. The southern slope indicated a U-shaped curve, with both ends possessing higher Sorensen dissimilarity indexes than the mid-elevational area (Fig. 5). In contrast, the northern slope indicated a roughly hump-shaped elevational pattern, with the peak of beta diversity occurring at approximately 2500–2800 m a.s.l. (Fig. 5). However, the distance decay of similarity was verified on both slopes, as suggested by the positive coefficients of the Mantel tests between dissimilarity and geographic distance (southern slope: *r* = 0.67, *P* = 0.002; northern slope: *r* = 0.90, *P* = 0.001; see Supplementary Fig. S9). With increasing elevation, the species composition of the assemblages on the two slopes became more similar, as suggested by the Sorensen dissimilarity index between the assemblages on the different slopes with similar elevations (standardized beta slope = −0.79; Fig. 6). At the regional scale, the southern slope as a whole possessed a higher value of beta diversity than the northern slope, as suggested by the Whittaker beta diversity index (southern slope: 2.82; northern slope: 2.33).

## Discussion

Mt. Taibai harbours diverse rodent fauna (30 species on a single mountain), which is often the case for an ecotone. Species can easily disperse between the distinct communities of an ecotone, thereby generating peaks in richness within the transitional zones^48^. Meanwhile, the southern slope of Mt. Taibai contains more rodent species than the northern slope. Higher rainfall and primary productivity on the southern slope may contribute to this pattern. Although the AMT does not differ significantly between the slopes, an environment with better vegetation and higher water availability should benefit both foraging and development. Tang *et al*. suggested that the southern slope of Mt. Taibai has higher woody plant species richness, which means potentially more niche space available for rodents^35^. Although not included in our analysis, historical factors, such as species dispersion and disturbance, may also be important. As mentioned before, the southern slope of Mt. Taibai is connected to neighbouring mountains, which may effectively act as species pools. The relatively high species richness observed in the lower elevational bands of the southern slope suggests the possibility of species dispersion. Conversely, the northern slope of Mt. Taibai is surrounded by rivers and villages. The rivers act as hard boundaries for most of the native rodent species, and the villages may have adverse effects on many rodents due to anthropogenic disturbance via occasional trapping, hunting and logging. Although we attempted to avoid those severely disturbed areas during our field survey, the effects of these disturbances on rodents, especially in the lowland area along the northern slope, cannot be completely excluded and deserve further investigation. Given these conditions, it is not surprising to find that the southern slope possesses more rodent species.

According to the survey, the northern slope of Mt. Taibai was dominated by Palaearctic species, and the southern slope was dominated by Oriental species. This pattern indicates that in terms of rodents, Mt. Taibai forms a portion of the boundary between the Palaearctic and the Oriental realms. A survey based on amphibian distribution suggests that the boundary between the two realms in western China is a broad transitional belt rather than a line^49^. Our results support this opinion. The rodent fauna belonging to the two realms is mixed on both slopes of Mt. Taibai (Fig. 2). Although the northern slope is dominated by Palaearctic species and the southern slope is dominated by Oriental species, each slope possesses several species from both realms. Interestingly, the middle part of the northern slope of Mt. Taibai harbours more Oriental rodent species than the upper part, suggesting that the dispersal distance cannot fully explain the spatial distribution of rodent species in this area.

Our results indicate that species richness patterns along the two slopes of Mt. Taibai are determined by different sets of factors. Again, the roles of the geometric constraints in shaping richness patterns vary between gradients. The prediction that a larger area harbours more species was only supported by the larger-ranged species along the northern slope. Species richness along the southern slope was negatively related to area. Similarly, the MDE is more significant on the northern slope. A plausible explanation is that the lower part of the southern slope contains many smaller-range species (Supplementary Table S1). In summary, geometric constraints play important roles along the northern slope, while environmental factors (i.e., AMT) play important roles along the southern slope. Similar to previous studies^10, 19^, our results suggest that different species groups are correlated with different factors, with larger-ranged species more associated with the MDE and smaller-ranged species more associated with environmental factors. However, these results should be considered exploratory rather than definitive, as we only included a small subset of climatic factors in this study and the effect of multicollinearity among variables was not fully avoided. Other environmental factors, such as annual range of temperature, plant species richness and habitat heterogeneity, may also contribute to the observed richness patterns and deserve further study.

While climatic factors are thought to be important in shaping richness patterns, the underlying mechanisms remain controversial. Two main candidate mechanisms related to climatic factors are the productivity hypothesis and the ambient energy hypothesis^16^. The productivity hypothesis (or the “energy-richness hypothesis”) states that the effects of water and energy availability occur via trophic cascades^50–52^. Higher primary production caused by higher water-energy availability can sustain more herbivores, and more herbivores should, in turn, sustain more predators. The ambient energy hypothesis (or the “physiological tolerance hypothesis”) states that the distribution of species is mainly determined by physiological constraints, such as frost tolerance or thermoregulatory limits^15, 17^. If the productivity hypothesis is correct, we should expect a positive association between richness and primary productivity, as well as between richness and water availability. Our results do not support this hypothesis, as the effect of primary production on elevational richness patterns is weak. In our study, the southern slope receives much more rainfall than the northern slope as a consequence of the hampering effect of the mountain body. This difference suggests that the organisms on the southern slope should be less, if at all, constrained by water availability. Therefore, the effects of other climatic factors (e.g., temperature) may override the influence of precipitation. Similar to the findings of Wu *et al*.^19^, the richness along the northern slope is negatively related to precipitation, which suggests that water availability should not be a significant limiting factor for rodent diversity in this area. If the ambient energy hypothesis is correct, we should expect higher diversity in the warmer area, which is the case for the southern slope. Our results seem to support the ambient energy hypothesis rather than the productivity hypothesis. This finding is reasonable since our study was conducted in a region with relatively high productivity, so that ambient energy may play a more important role in generating the richness pattern.

In terms of species turnover, the distance decay of similarity in communities is generally supported, which is also true for the between-slope comparison. This result suggests that dispersal limitation, either caused by environmental or geographic barriers, greatly regulates the rodent species distribution pattern in this region. However, the two slopes of Mt. Taibai show distinct elevational patterns of beta diversity. The difference in the fauna between the slopes may partly explain this phenomenon. It has been suggested that tropical species are more likely to be specialists while species at high latitudes tend to have larger ranges, which partly explains why beta diversity generally increases with decreasing latitude^53, 54^. In our study system, the lowland area of the southern slope is dominated by many Oriental species. Of these Oriental species, 14 species are only recorded in the lowest elevational band, thus generating a rapid species turnover at the lower end of the gradient. On the other hand, the hump-shaped pattern found on the northern slope may reflect a transition between mid-elevation and highland faunas^55, 56^. On the higher end of the northern slope (above 3200 m a.s.l.), three species dominate the assemblages (*Eothenomys eva*, *Apodemus peninsulae* and *Ochotona thibetana*) and form a highland fauna that is distinct from the rest of the gradient. Meanwhile, the addition of species occurring at mid-elevations may also contribute to this phenomenon^7^, which is exactly the case in the present study. On a regional scale, the higher beta diversity along the southern slope may be related to the higher precipitation and the dominance of tropical species on this slope, while the role of environmental heterogeneity also deserves attention.

Our results indicate that even within the same mountain, organisms inhabiting different slopes may possess distinct distribution patterns, and the underlying mechanisms controlling this distribution may also differ. Although we only investigated a single mountain and the generality of our results remains unknown, our study clearly notes such a possibility. The differences in species composition, environmental conditions, as well as some historical factors between the different slopes may contribute to this finding. It will be useful to investigate the potential effects of the factors associated with slope aspect when comparing and interpreting the results of previous studies. More studies comparing diversity patterns between slopes are also needed.

## Methods

### Study area

The Qinling Mountains, which spread from west to east in Central China, act not only as a watershed between the Yangtze River and the Yellow River but also as an important physical obstacle for large-scale air mass movements. The Qinling Mountains themselves are also a world-famous biodiversity hotspot. In a biogeographical sense, the Qinling Mountains represent a transition between the Palaearctic and the Oriental realms^49^.

In this study, we focus on Mt. Taibai (107°22′–107°55′E, 33°45′–34°10′N), the highest peak of the Qinling Mountains. Mt. Taibai is characterized by a steep northern slope and a gentle southern slope. The climate of the northern slope is more continental than the southern slope, with the former having a higher annual range of temperature and a higher lapse rate of annual mean temperature^45^. Mt. Taibai has various temperate zones along the altitudinal gradient, including a warm temperate zone (800–1200 m a.s.l.), a mid-temperate zone (1201–2400 m a.s.l.), a cold temperate zone (2401–3400 m a.s.l.) and a sub-cold zone (3401–3767 m a.s.l.)^57^.

Mt. Taibai has an extremely diverse flora, with 1783 seed plant species, 325 bryophyte species and 110 fern species recorded^58^. The vegetation of Mt. Taibai shows a clear elevational pattern: (1) *Quercus variabilis* deciduous broad-leaf forest (northern slope: below 1200 m a.s.l.; southern slope: below 1400 m a.s.l.); (2) *Q. aliena* deciduous broad-leaf forest (northern slope: 1200–1650 m a.s.l.; southern slope: 1400–2050 m a.s.l.); (3) *Q. mongolica* deciduous broad-leaf forest (only found on the northern slope: 1650–2300 m a.s.l.); (4) *Betula albo-sinensis* deciduous broad-leaf forest (northern slope: 2300–2600 m a.s.l.; southern slope: 2250–2500 m a.s.l.); (5) *B. utilis* deciduous broad-leaf forest (northern slope: 2600–2950 m a.s.l.; southern slope: 2500–2850 m a.s.l.); (6) *Abies fargesii* coniferous forest (northern slope: 2900–3150 m a.s.l.; southern slope: 2850–3200 m a.s.l.); (7) *Larix chinensis* coniferous forest (northern slope: 3150–3400 m a.s.l.; southern slope: 3200–3430 m a.s.l.); and (8) *Rhododendron capitatum* shrub (northern slope: above 3400 m a.s.l.; southern slope: above 3430 m a.s.l.)^59^.

### Field survey

We performed this study in accordance with the guidelines of the American Society of Mammalogists for the use of wild animals in research^60^ and under the approval from the Animal Ethics Committee at the Institute of Zoology, Chinese Academy of Sciences. To investigate the elevational patterns of rodent species richness, we conducted a field survey from June to September 2007. Rodents were sampled at 57 sites along two gradients (28 sites on the northern slope and 29 sites on the southern slope, with a minimum interval of 200 m between neighbouring sites; Fig. 1). The northern slope gradient ranged from 1000 to 3700 m a.s.l., and the southern slope gradient ranged from 1250 to 3650 m a.s.l. The regions at lower elevations were not sampled because they were either not available due to connection with neighbouring mountains (the southern slope) or they were highly disturbed by anthropogenic activities (the northern slope). The western part of the northern slope was not sampled due to low accessibility. At each site, we placed 200 locally made wire cages (at 5-m intervals between neighbouring traps) along 4 transects, with a minimum distance of 30 m between transects. Cages were baited with fried peanuts, which, to our knowledge, are highly attractive to most rodent species in this region. We checked the cages every morning and rebaited them when necessary. All captured individuals were identified to species, sexed, weighed and then released. We used species accumulation curves (see Supplementary Fig. S10) to evaluate if the sampling was sufficient at each site. Trapping at each site lasted 4–5 days until the species accumulation curves plateaued. Visual observations were also conducted at each site to record the arboreal species such as squirrels because cages are less efficient at capturing these species^19^. During visual observations, we only recorded the presence of each rodent species at each site and did not attempt to record its relative frequency. Despite our efforts, it is unlikely that all rodent species in the natural habitats were recorded due to time and space limitations. To reduce the possible influences of inadequate sampling and seasonal effects, we supplemented the data with records from a previous investigation carried out between 2005 and 2006^58^. Only those records with clear spatial distribution patterns were added to our data. The biogeographic realm of each recorded species was classified according to Zhang^61^.

To document the elevational distribution of richness, both gradients were divided into several 250-m elevational bands (e.g., 1250–1499 m, 1500–1749 m). We used range interpolation to address the gaps in the species ranges, i.e., a species was assumed to exist at an elevational band if it was found at both higher and lower elevations. This assumption may not hold true; if so, range interpolation may bias the actual distribution patterns and increase the possibility of generating a hump-shaped richness curve. However, it has been suggested that on such a spatiotemporal scale, the disconnected distribution is more likely a result of sampling incompleteness rather than true gaps in distribution^27, 62^. The range size of each species used in the MDE simulation was represented by “n × 250 m”, where “n” was the number of elevational bands where the species was recorded or was assumed to occur^63^.

### Climate and productivity

We included two highly documented climatic factors in our analysis: AMT and AMP. Although other climatic factors may also contribute to the richness patterns, we decided to exclude them to reduce multicollinearity. We estimated AMT for each elevational band on the southern and northern slopes of Mt. Taibai from empirical formulas (the northern slope: AMT = −0.0049 × Altitude + 17.9; the southern slope: AMT = −0.0037 × Altitude + 14.5; both in units of centigrade) provided by Tang *et al*.^35^. These formulas were based on long-term *in situ* measurements of temperature from HOBO microloggers (Onset Corporation, Bourne, Massachusetts, USA) and were proven to adequately represent the long-term elevational patterns of temperature in this area^35^. We obtained the values of AMP from the dataset (grid data with a 500 × 500 m resolution, representing mean values of multi-year recordings obtained from local weather stations) provided by The Data Center for Resources and Environmental Sciences, Chinese Academy of Sciences (RESDC) (http://www.resdc.cn). For each gradient, the AMP for each elevational band was calculated as the mean value of the grids within each band.

We used the EVI as a surrogate for aboveground net primary productivity. Compared with the normalized difference vegetation index (NDVI), EVI is more sensitive to topographic conditions^64^ and can effectively account for atmospheric contamination^12, 65^. Using the dataset (grid data with a 500 × 500 m resolution) derived from the Moderate-resolution Imaging Spectroradiometer (MODIS) in the Geographical Information Monitoring Cloud Platform (http://www.dsac.cn), we calculated the averaged EVI between June 1^st^ and September 30^th^ in 2007 for each elevational band gradient in ArcGIS 10.1 (Environmental System Research Institute Inc., Redlands, California, USA; http://www.esri.com).

### Geometric factors

To explore the relationship between area and species richness, we calculated the surface area for each elevational band per gradient. The calculations were conducted in ArcGIS 10.1 based on 30-m digital elevation models (DEM) obtained from https://wist.echo.nasa.gov/. To assess the contribution of the MDE to the elevational patterns of species richness, we used RangeModel 5^66^ to generate the mean predicted elevational patterns of species richness for each gradient and their 95% confidence intervals. The calculation of the null MDE distribution for each gradient was based on 50,000 Monte Carlo simulations (sampling with replacement). The predicted richness was then used in a linear regression against empirical richness to test the goodness of fit of the null MDE model. We also included the predicted values of the null MDE models as an explanatory factor in our multivariate analyses.

### Data analysis

We first conducted polynomial regressions to explore the shapes of the richness-elevation curves for each slope aspect (to determine if the relationship is more likely to be linear, quadratic or cubic). We used AIC_c_ to select the best model for each gradient. The AIC is based on the Kullback-Leibler information and reflects a balance between goodness of fit and parsimony, or a balance between under- and over-fit models^67^. The AIC_c_ is a small-sample version of AIC and should be used when the sample size is relatively small compared to the number of parameters^67^. Similar analyses were also performed on the number of Oriental species and Palaearctic species to explore the relationship between species composition and elevation on each slope.

We conducted multiple regressions to identify the best multivariate model in explaining the elevational pattern of species richness for each gradient. Multiple OLS regressions were carried out for each combination of explanatory factors; thus, 31 different models were generated for each gradient. We computed the AIC_c_ for each model and used these values as a criterion to rank the models in each gradient. However, it may be problematic to determine a “best” model simply using AIC_c_ scores, especially when several models possess nearly equal AIC_c_ values. We, therefore, used the impact factor of each explanatory variable to measure its relative importance in shaping the elevational patterns of species richness in each gradient. Impact factors are calculated as the sum of the weights of the models that include a given variable and can be viewed as posterior probabilities over the whole set of models. We defined an explanatory factor as “important” if its impact factor was ≥0.80^12^. Spatial autocorrelation generally exists in geographical data because measurements from nearby sites are often not independent. However, it is not suitable to conduct spatial autoregressive analysis with five explanatory factors due to the small sample size (10 and 11 samples on the southern and northern slopes, respectively); therefore, we did not report *p*-values for the multiple regressions.

To explore the effects of a species range on richness patterns and the underlying mechanisms, we divided the total species equally into two groups: “larger-ranged” (the half of the species with elevational ranges above the median range size) and “smaller-ranged” (the other half of the species with elevational ranges below the median range size). We repeated all analyses described above (except for the analyses on species composition) for these two groups separately along each gradient.

We employed the widely used Sorensen dissimilarity index to measure species turnover between a pair of elevational bands. The Sorensen dissimilarity index is defined as S = (b + c)/(2a + b + c), where a, b and c represent the number of species shared by both sites, the number of species only found at one site and the number of species only found at the other site, respectively^68^. This index takes a value ranging from 0 to 1 which represents a continuum from no species shared to complete similarity between assemblages. We calculated the Sorensen dissimilarity index for (1) the paired elevational bands along each gradient (e.g., 2000–2249 m a.s.l. *vs*. 2250–2499 m a.s.l., 2250–2499 m a.s.l. *vs*. 2500–2749 m a.s.l.), (2) each pair of elevational bands within each gradient (i.e., a Sorensen dissimilarity matrix for each slope), and (3) each pair of elevational bands with similar elevations on different slopes (10 pairs of bands altogether). We generated the geographic distance matrix using the Geographic Distance Matrix Generator (freely available at http://biodiversityinformatics.amnh.org/open_source/gdmg/)^69^. To evaluate the distance decay of similarity, we used the “mantel” function in the R package “ecodist”^70^ (based on 1000 permutations) to carry out simple Mantel tests between the Sorensen dissimilarity matrix and the geographic distance matrix. To evaluate the relationship between species turnover and elevation, we conducted polynomial regressions on the Sorensen dissimilarity against elevation. For comparison on a regional scale, we also calculated the Whittaker beta diversity index for each slope as a whole. The Whittaker index was calculated as W = (s/α) − 1, where s is the total number of species found in the whole gradient, and α is the average number of species recorded at each site^71^.

We used SAM 4.0^72^ (freely available at http://www.ecoevol.ufg.br/sam/) to perform OLS regressions and multi-model inference. Polynomial regressions were conducted in PAST 3.12^73^ (freely available at http://folk.uio.no/ohammer/past/). We used SPSS 19.0 (SPSS Inc., Chicago, Illinois, USA) to plot the figures.

## Electronic supplementary material

Supplemental Information

## References

[1] Brown JH (2001). Mammals on mountainsides: Elevational patterns of diversity. Glob. Ecol. Biogeogr..

[2] McCain CM (2005). Elevational gradients in diversity of small mammals. Ecology.

[3] Körner C (2007). The use of ‘altitude’ in ecological research. Trends Ecol. Evol..

[4] Körner C (2017). Why are there global gradients in species richness? mountains might hold the answer. Trends Ecol. Evol..

[5] Stevens GC (1992). The elevational gradient in altitudinal Range: An extension of Rapoport’s latitudinal rule to altitude. Am. Nat..

[6] Rahbek C (1995). The elevational gradient of species richness: a uniform pattern?. Ecography.

[7] Heaney LR (2001). Small mammal diversity along elevational gradients in the Philippines: An assessment of patterns and hypotheses. Glob. Ecol. Biogeogr..

[8] Rickart EA (2001). Elevational diversity gradients, biogeography and the structure of montane mammal communities in the intermountain region of North America. Glob. Ecol. Biogeogr..

[9] McCain CM (2004). The mid-domain effect applied to elevational gradients: species richness of small mammals in Costa Rica. J. Biogeogr..

[10] Fu C (2006). Elevational of frog and endemic in richness patterns species richness the Hengduan Mountains, area and China: geometric constraints, climate effects. Ecography.

[11] Kluge J, Kessler M, Dunn RR (2006). What drives elevational patterns of diversity? A test of geometric constraints, climate and species pool effects for pteridophytes on an elevational gradient in Costa Rica. Glob. Ecol. Biogeogr..

[12] Rowe RJ (2009). Environmental and geometric drivers of small mammal diversity along elevational gradients in Utah. Ecography.

[13] Herzog SK, Kessler M, Bach K (2005). The elevational gradient in Andean bird species richness at the local scale: a foothill peak and a high-elevation plateau. Ecography.

[14] Connell JH (1961). The influence of interspecific competition and other factors on the distribution of the barnacle *Chthamlus Stellatus*. Ecology.

[15] Currie DJ (1991). Energy and large-scale patterns of animal - and plant - species richness. Am. Nat..

[16] Hawkins BA (2003). Energy, water, and broad-scale geographic patterns of species richness. Ecology.

[17] Currie DJ (2004). Predictions and tests of climate-based hypotheses of broad-scale variation in taxonomic richness. Ecol. Lett..

[18] Bailey SA (2004). Primary productivity and species richness: relationships among functional guilds, residency groups and vagility classes at multiple spatial scales. Ecography.

[19] Wu Y (2013). What drives the species richness patterns of non-volant small mammals along a subtropical elevational gradient?. Ecography.

[20] McCain CM (2007). Area and mammalian elevational diversity. Ecology.

[21] Preston FW (1962). The canonical distibution of commonness and rarity: Part I. Ecology.

[22] MacArthur, R. H. & Wilson, E. O. *The theory of island biogeography*. *Monographs in Population Biology* **1**, (Princeton University Press, 1967).

[23] Rosenzweig, M. L. *Species Diversity in Space and Time*. (Cambridge University Press, 1995).

[24] Colwell RK, Hurtt GC (1994). Nonbiological gradients in species richness and a spurious Rapoport effect. Am. Nat..

[25] Colwell RK, Lees DC (2000). The mid-domain effect: geometric constraints on the geography of species richness. Trends Ecol. Evol..

[26] Lees DC, Kremen C, Andriamampianina L (1999). A null model for species richness gradients: bounded range overlap of butterflies and other rainforest endemics in Madagascar. Biol. J. Linn. Soc..

[27] Colwell RK, Rahbek C, Gotelli NJ (2004). The mid-domain effect and species richness patterns:what have we learned so far?. Am. Nat..

[28] Laurie H, Silander JA (2002). Geometric constraints and spatial pattern of species richness: critique of range-based null models. Divers. Distrib..

[29] Zapata FA, Gaston KJ, Chown SL (2003). Mid-domain models of species richness gradients: assumptions, methods and evidence. J. Anim. Ecol..

[30] Jetz W, Rahbek C (2001). Geometric constraints explain much of the species. Proc. Natl. Acad. Sci. USA.

[31] Sanders NJ (2002). Elevational gradients in ant species richness: area, geometry, and Rapoport’s rule. Ecography.

[32] Watkins JE, Cardelús C, Colwell RK, Moran RC (2006). Species richness and distribution of ferns along an elevational gradient in Costa Rica. Am. J. Bot..

[33] Gallardo-Cruz A, Pérez-García EA, Meave JA (2009). β-Diversity and vegetation structure as influenced by slope aspect and altitude in a seasonally dry tropical landscape. Landsc. Ecol..

[34] Armesto JJ, Martinez JA (1978). Relations between vegetation structure and slope aspect in the mediterranean region of Chile. J. Ecol..

[35] Tang Z, Fang J, Zhang L (2004). Patterns of woody plant species diversity along environmental gradients on Mt.Taibai, Qinling Mountains. Biodivers. Sci..

[36] Wang L, Wei S, Horton R, Shao M (2011). Effects of vegetation and slope aspect on water budget in the hill and gully region of the Loess Plateau of China. Catena.

[37] Kilgore MJ, Fairbanks WS (1997). Winter habitat selection by reintroduced pronghorn on Antelope Island, Great Salt Lake, Utah. Gt. Basin Nat..

[38] Harvey DS, Weatherhead PJ (2006). Hibernation site selection by eastern massasauga rattlensakes (*Sistrurus catenatus catenatus*) near their northern range limit. J. Herpetol..

[39] Currylow AF, MacGowan BJ, Williams RN (2013). Hibernal thermal ecology of eastern box turtles within a managed forest landscape. J. Wildl. Manage..

[40] Koleff P, Gaston KJ, Lennon JJ (2003). Measuring beta diversity for presence-absence data. J. Anim. Ecol..

[41] Wilson MVM, Shmida A (1984). Measuring beta diversity with presence-absence data. J. Ecol..

[42] Koleff P, Gaston KJ (2001). Latitudinal gradients in diversity: real patterns and random models. Ecography.

[43] Wen Z (2016). Dispersal, niche, and isolation processes jointly explain species turnover patterns of nonvolant small mammals in a large mountainous region of China. Ecol. Evol..

[44] Mena JL, Vázquez-Domínguez E (2005). Species turnover on elevational gradients in small rodents. Glob. Ecol. Biogeogr..

[45] Tang Z, Fang J (2006). Temperature variation along the northern and southern slopes of Mt. Taibai, China. Agric. For. Meteorol..

[46] Nekola JC, White PS (1999). The distance decay of similarity in biogeography and ecology. J. Biogeogr..

[47] Soininen J, McDonald R, Hillebrand H (2007). The distance decay of similarity in ecological communities. Ecography.

[48] Lomolino MV (2001). Elevation gradients of species-density: historical and prospective views. Glob. Ecol. Biogeogr..

[49] Chen L, Song Y, Xu S (2008). Boundary of palaearctic and oriental realms in western China. Prog. Nat. Sci..

[50] Hutchinson GE (1959). Homage to Santa Rosalia or why are there so many kinds of animals?. Am. Nat..

[51] Connell JH, Orias E (1964). The ecological regulation of species diversity. Am. Nat..

[52] Hurlbert AH, Haskell JP (2003). The effect of energy and seasonality on avian species richness and community composition. Am. Nat..

[53] Stevens GC (1989). The latitudinal gradient in geographical range: how so many species coexist in the tropics?. Am. Nat..

[54] Jankowski JE, Ciecka AL, Meyer NY, Rabenold KN (2009). Beta diversity along environmental gradients: implications of habitat specialization in tropical montane landscapes. J. Anim. Ecol..

[55] Patterson BD, Stotz DF, Solari S, Fitzpatrick JW, Pacheco V (1998). Contrasting patterns of elevational zonation for birds and mammals in the Andes of southeastern Peru. J. Biogeogr..

[56] Nor SMD (2001). Elevational diversity patterns of small mammals on Mount Kinabalu, Sabah, Malaysia. Glob. Ecol. Biogeogr..

[57] Chen M (1992). The regionalization of vertical temperature zones in Qinling Montain. J. Northwest Univ. (Natural Sci. Ed.).

[58] Ren, Y., Liu, M., Tian, L., Tian, X. & Li, Z. *Biodiversity, Conservation and Management of Taibaishan Nature Reserve*. (China Forestry Publishing House, 2006).

[59] Tang, Z. Patterns of plant species diversity in the Qinling Montains, Central China. (Peking University, 2003).

[60] Sikes RS, Gannon WL (2011). & Animal Care and Use Committee of the American Society of Mammalogists. Guidelines of the American Society of Mammalogists for the use of wild mammals in research. J. Mammal..

[61] Zhang, R. Z. *Zoogeography of China*. (Science Press, 1999).

[62] Grytnes JA, Vetaas OR (2002). Species richness and altitude: a comparison between null models and interpolated plant species richness along the Himalayan altitudinal gradient, Nepal. Am. Nat..

[63] Pan X (2016). Elevational pattern of bird species richness and its causes along a central Himalaya gradient, China. PeerJ.

[64] Matsushita B, Yang W, Chen J, Onda Y, Qiu G (2007). Sensitivity of the Enhanced Vegetation Index (EVI) and Normalized Difference Vegetation Index (NDVI) to topographic effects: a case study in high-density cypress forest. Sensors.

[65] Huete AR, Liu HQ, Batchily K, Van Leeuwen W (1997). A comparison of vegetation indices over a global set of TM images for EOS-MODIS. Remote Sens. Environ..

[66] Colwell, R. K. RangeModel: A Monte Carlo simulation tool for assessing geometric constraints on species richness.Version 5. User’s Guide and application published at: http://viceroy.eeb.uconn.edu/rangemodel, Available at: http://viceroy.eeb.uconn.edu/rangemodel (2006).

[67] Burnham KP, Anderson DR (2004). Multimodel inference: Understanding AIC and BIC in model selection. Sociol. Methods Res..

[68] Baselga A (2010). Partitioning the turnover and nestedness components of beta diversity. Glob. Ecol. Biogeogr..

[69] Ersts, P. J. Geographic Distance Matrix Generator (version 1.2.3). *American Museum of Natural History, Center for Biodiversity and Conservation*. 1–4 Available at: http://biodiversityinformatics.amnh.org/open_source/gdmg (2014).

[70] Goslee SC, Urban DL (2007). The ecodist package for dissimilarity-based analysis of ecological data. J. Stat. Softw..

[71] Whittaker RH (1960). Vegetation of the Siskiyou Mountains, Oregon and California. Ecol. Monogr..

[72] Rangel TF, Diniz-Filho JAF, Bini LM (2010). SAM: A comprehensive application for Spatial Analysis in Macroecology. Ecography.

[73] Hammer Ø, Harper DATaT, Ryan PD (2001). PAST: Paleontological Statistics Software Package for Education and Data Analysis. Palaeontol. Electron..

