# Supplementary material for: Different elevational patterns of rodent species richness between the southern and northern slopes of a mountain
Source: Sci Rep. 2017 Aug 18;7:8743. doi: 10.1038/s41598-017-09274-2 (PMC5562761; doi:10.1038/s41598-017-09274-2)

Different elevational patterns of rodent species richness between the southern and northern slopes of a moutain

Ling-Ying Shuai[1,2], Chun-Lei Ren[1], Wen-Bo Yan[3], Yan-Ling Song[1] and Zhi-Gao Zeng[1,*]

[1] *Key Laboratory of Animal Ecology and Conservation Biology, Institute of Zoology, Chinese Academy of Sciences, Beijing 100101, China*
[2] *College of Life Sciences, Huaibei Normal University, Huaibei 235000, China*
[3] *Bio-resources Key Laboratory of Shannxi Province, Shannxi Sci-Tech University, Hanzhong 723001, China*

* Corresponding author e-mail: zengzhg@ioz.ac.cn

Supplementary material

Supplementary Table S1. Elevational distribution (m a.s.l.) of rodent species on Mt. Taibai, China. *: Oriental species. #: Species added from Ren et al (2006).

Species	Southern slope		Northern slope	
	Minimum	Maximum	Minimum	Maximum
**Sciuridae**				
*Callosciurus erythraeus**	1280	1280	Not found	
*Tamiops swinhoei**	1320	1320	2800	2800
*Dremomys pernyi**	1280	1320	1100	2150
Sciurotamias davidianus	1320	1320	1350	2600
Eutamias sibiricus	2550	2850	2020	2900
**Petauristidae**				
Trogopterus xanthipes#	1280	1280	Not found	
*Petaurista alborufus**	1320	1320	Not found	
Petaurista elegans,#*	1280	1600	Not found	
**Cricetidae**				
*Eothenomys eva**	1300	3600	1840	3600
Eothenomys inez	1800	3400	1100	1650
*Eothenomys melanogaster**	1280	2300	Not found	
Myospalax smithi	Not found		2300	2300
Microtus oeconomus	2300	2800	2100	2700
Cricetulus triton	1320	1320	1150	2300
**Rhizomyidae**				
*Rhizomys sinensis**	1320	2650	Not found	
**Platacanthomyidae**				
Typhomys cinereus,#*	1550	1550	Not found	
*Vernaya fulva**	1320	2100	1800	2020
**Muridae**				
Apodemus agrarius	1320	1320	1100	1700
Apodemus peninsulae	1320	3400	1000	3700
*Apodemus chevrieri**	1320	2800	1100	2700
*Rattus flavipectus**	1280	1320	1000	1300
Rattus nitidus,#*	1280	3050	Not found	
*Niviventer fulvescens**	1280	1320	Not found	
*Niviventer niviventer**	1280	2400	1350	2800
*Niviventer coxingi**	1280	1320	Not found	
*Leopoldamys edwardsi**	1320	1800	Not found	
**Zapodidae**				
Eozapus setchuanus	1400	3050	1650	2700
Sicista concolor	1320	3050	1650	3050
**Hystricidae**				
Hystrix hodgsoni,#*	1280	1550	Not found	
**Ochotonidae**				
Ochotona thibetana	3050	3650	2800	3700

Supplementary Table S2. Polynomial regressions on rodent species richness (interpolated) against elevation for different slopes of Mt. Taibai, China. Bold numbers suggest the best polynomial regression model (with lowest AIC_c value). Larger-ranged species are the 50% of species with ranges above median range size, and smaller-ranged species are the other species. * $p < 0.05$; ** $p < 0.01$.

	First-order model		Second-order model		Third-order model	
	r^2	AIC_c	r^2	AIC_c	r^2	AIC_c
Southern slope						
Total species	0.843**	57.61	0.867**	54.13	0.956**	**30.53**
Larger-ranged species	0.828**	23.67	0.975**	**12.61**	0.977**	18.40
Smaller-ranged species	0.369	98.90	0.673*	58.27	0.909**	**29.40**
Northern slope						
Total species	0.448*	61.86	0.873**	22.37	0.931**	**21.71**
Larger-ranged species	0.187	59.6	0.853**	19.21	0.958**	**17.47**
Smaller-ranged species	0.838**	**7.54**	0.838*	11.46	0.842*	16.65

Supplementary Table S3. Summary for multiple regressions on total rodent species richness (interpolated) along the southern slope of Mt. Taibai. N: number of variables; r^2: coefficient of determination; C.N.: condition number; AIC_c: model AIC_c score; D. A.: model AIC_c difference; W_i: model Akaike weight. Five explanatory variables: Area, EVI (enhanced vegetation index), AMT (annual mean temperature), AMP (annual mean precipitation) and MDE (mid-domain effect).

Model #	Variables	N	r^2	C.N.	AIC_c	D.A.	W_i
10	Area, AMT	2	0.971	2.387	43.999	0	0.898
13	Area, AMT, MDE	3	0.979	3.324	49.938	5.939	0.046
2	Area, EVI	2	0.932	2.825	52.472	8.474	0.013
11	Area, AMT, AMP	3	0.971	12.686	52.771	8.772	0.011
3	Area, EVI, AMT	3	0.971	16.108	52.995	8.997	0.01
15	Area, AMP, MDE	3	0.97	4.482	53.271	9.272	0.009
25	AMT	1	0.843	1	54.844	10.846	0.004
26	AMT, AMP	2	0.91	8.641	55.264	11.266	0.003
18	EVI, AMT	2	0.902	10.858	56.111	12.113	0.002
14	Area, AMP	2	0.896	2.936	56.76	12.761	0.002
7	Area, EVI, AMP	3	0.947	9.623	59.051	15.053	<0.001
28	AMT, MDE	2	0.867	1.252	59.153	15.155	<0.001
17	EVI	1	0.736	1	60.014	16.015	<0.001
19	EVI, AMT, AMP	3	0.939	13.327	60.426	16.427	<0.001
27	AMT, AMP, MDE	3	0.937	8.843	60.74	16.741	<0.001
9	Area, EVI, MDE	3	0.933	3.582	61.39	17.391	<0.001
29	AMP	1	0.696	1	61.423	17.425	<0.001
12	Area, AMT, AMP, MDE	4	0.983	20.886	62.529	18.531	<0.001
24	EVI, MDE	2	0.805	1.433	63.001	19.003	<0.001
6	Area, EVI, AMT, MDE	4	0.982	19.823	63.366	19.367	<0.001
21	EVI, AMT, MDE	3	0.903	15.94	65.041	21.042	<0.001
22	EVI, AMP	2	0.736	8.18	66.012	22.013	<0.001
30	AMP, MDE	2	0.713	1.236	66.877	22.879	<0.001
4	Area, EVI, AMT, AMP	4	0.972	23.354	67.738	23.74	<0.001
8	Area, EVI, AMP, MDE	4	0.97	19.145	68.229	24.231	<0.001
23	EVI, AMP, MDE	3	0.833	10.481	70.462	26.463	<0.001
1	Area	1	0.151	1	71.708	27.709	<0.001
31	MDE	1	0.003	1	73.322	29.323	<0.001
20	EVI, AMT, AMP, MDE	4	0.941	19.911	74.968	30.969	<0.001
16	Area, MDE	2	0.201	1.973	77.105	33.107	<0.001
5	Area, EVI, AMT, AMP, MDE	5	0.989	24.493	87.923	43.924	<0.001

Supplementary Table S4. Summary for multiple regressions on total rodent species richness (interpolated) along the northern slope of Mt. Taibai. N: number of variables; r^2: coefficient of determination; C.N.: condition number; AIC_c: model AIC_c score; D. A.: model AIC_c difference; W_i: model Akaike weight. Five explanatory variables: Area, EVI (enhanced vegetation index), AMT (annual mean temperature), AMP (annual mean precipitation) and MDE (mid-domain effect).

Model #	Variables	N	r^2	C.N.	AIC_c	D.A.	W_i
30	AMP, MDE	2	0.972	1.133	31	0	0.774
15	Area, AMP, MDE	3	0.981	4.175	34.076	3.076	0.166
23	EVI, AMP, MDE	3	0.974	3.196	37.572	6.571	0.029
27	AMT, AMP, MDE	3	0.972	4.323	38.322	7.321	0.02
12	Area, AMT, AMP, MDE	4	0.986	6.496	41.98	10.979	0.003
8	Area, EVI, AMP, MDE	4	0.985	8.629	42.277	11.277	0.003
20	EVI, AMT, AMP, MDE	4	0.984	14.239	43.002	12.002	0.002
16	Area, MDE	2	0.906	2.039	44.444	13.444	<0.001
28	AMT, MDE	2	0.899	1.179	45.156	14.155	<0.001
1	Area	1	0.833	1	45.48	14.48	<0.001
2	Area, EVI	2	0.874	4.414	47.575	16.574	<0.001
6	Area, EVI, AMT, MDE	4	0.974	12.292	48.61	17.609	<0.001
24	EVI, MDE	2	0.861	1.396	48.728	17.728	<0.001
3	Area, EVI, AMT	3	0.922	11.602	49.636	18.636	<0.001
13	Area, AMT, MDE	3	0.92	5.433	49.946	18.946	<0.001
10	Area, AMT	2	0.842	2.967	50.125	19.124	<0.001
14	Area, AMP	2	0.838	2.47	50.387	19.387	<0.001
7	Area, EVI, AMP	3	0.909	5.409	51.321	20.321	<0.001
9	Area, EVI, MDE	3	0.906	7.546	51.678	20.678	<0.001
21	EVI, AMT, MDE	3	0.904	8.169	51.958	20.957	<0.001
11	Area, AMT, MDE	3	0.891	5.282	53.338	22.338	<0.001
31	MDE	1	0.596	1	55.203	24.203	<0.001
17	EVI	1	0.541	1	56.591	25.59	<0.001
29	AMP	1	0.496	1	57.618	26.618	<0.001
25	AMT	1	0.448	1	58.617	27.616	<0.001
5	Area, EVI, AMT, AMP, MDE	5	0.986	39.733	60.294	29.293	<0.001
4	Area, EVI, AMT, AMP	4	0.924	31.079	60.323	29.323	<0.001
22	EVI, AMP	2	0.582	2.904	60.797	29.796	<0.001
19	EVI, AMT, AMP	3	0.771	11.372	61.498	30.498	<0.001
18	EVI, AMT	2	0.554	6.608	61.526	30.525	<0.001
26	AMT, AMP	2	0.504	4.256	62.688	31.688	<0.001

Supplementary Table S5. Summary for multiple regressions on larger-ranged rodent species richness (interpolated) along the southern slope of Mt. Taibai. N: number of variables; r^2: coefficient of determination; C.N.: condition number; AIC_c: model AIC_c score; D. A.: model AIC_c difference; W_i: model Akaike weight. Five explanatory variables: Area, EVI (enhanced vegetation index), AMT (annual mean temperature), AMP (annual mean precipitation) and MDE (mid-domain effect).

Model #	Variables	N	r^2	C.N.	AIC_c	D.A.	W_i
28	AMT, MDE	2	0.977	1.206	30.27	0	0.572
30	AMP, MDE	2	0.971	1.195	32.594	2.324	0.179
15	Area, AMP, MDE	3	0.986	4.551	33.904	3.634	0.093
24	EVI, MDE	2	0.966	1.369	33.923	3.653	0.092
27	AMT, AMP, MDE	3	0.983	8.786	35.995	5.725	0.033
13	Area, AMT, MDE	3	0.979	3.347	38.437	8.167	0.01
21	EVI, AMT, MDE	3	0.977	15.016	39.188	8.918	0.007
23	EVI, AMD, MDE	3	0.976	10.034	39.497	9.227	0.006
17	EVI	1	0.892	1	39.622	9.353	0.005
2	Area, EVI	2	0.918	2.825	42.819	12.549	0.001
9	Area, EVI, MDE	3	0.966	3.627	42.892	12.622	0.001
25	AMT	1	0.828	1	44.23	13.96	<0.001
10	Area, AMT	2	0.905	2.387	44.258	13.989	<0.001
18	EVI, AMT	2	0.902	10.858	44.648	14.379	<0.001
29	AMP	1	0.815	1	44.96	14.69	<0.001
22	EVI, AMP	2	0.895	8.18	45.325	15.055	<0.001
14	Area, AMP	2	0.855	2.936	48.562	18.292	<0.001
12	Area, AMT, AMP, MDE	4	0.987	20.619	48.57	18.301	<0.001
8	Area, EVI, AMP, MDE	4	0.986	17.199	48.901	18.631	<0.001
1	Area	1	0.7	1	49.821	19.552	<0.001
26	AMT, AMP	2	0.834	8.641	49.907	19.637	<0.001
11	Area, AMT, AMP	3	0.929	12.686	50.448	20.179	<0.001
7	Area, EVI, AMP	3	0.928	9.623	50.515	20.245	<0.001
20	EVI, AMT, AMP, MDE	4	0.984	18.504	50.605	20.335	<0.001
3	Area, EVI, AMT	3	0.918	16.108	51.797	21.527	<0.001
6	Area, EVI, AMT, MDE	4	0.979	18.814	53.424	23.154	<0.001
19	EVI, AMT, AMP	3	0.902	13.327	53.629	23.36	<0.001
16	Area, MDE	2	0.706	1.924	55.597	25.328	<0.001
31	MDE	1	0.3	1	58.284	28.014	<0.001
4	Area, EVI, AMT, AMP	4	0.935	23.354	64.535	34.265	<0.001
5	Area, EVI, AMT, AMP, MDE	5	0.987	24.98	78.228	47.959	<0.001

Supplementary Table S6. Summary for multiple regressions on smaller-ranged rodent species richness (interpolated) along the southern slope of Mt. Taibai. N: number of variables; r^2: coefficient of determination; C.N.: condition number; AIC_c: model AIC_c score; D. A.: model AIC_c difference; W_i: model Akaike weight. Five explanatory variables: Area, EVI (enhanced vegetation index), AMT (annual mean temperature), AMP (annual mean precipitation) and MDE (mid-domain effect).

Model #	Variables	N	r^2	C.N.	AIC_c	D.A.	W_i
10	Area, AMT	2	0.96	2.387	39.126	0	0.829
14	Area, AMP	2	0.934	2.936	44.059	4.933	0.07
3	Area, EVI, AMT	3	0.97	16.108	45.249	6.123	0.039
11	Area, AMT, AMP	3	0.968	12.686	45.883	6.757	0.028
13	Area, AMT, MDE	3	0.967	2.833	46.138	7.012	0.025
2	Area, EVI	2	0.876	2.825	50.41	11.284	0.003
7	Area, EVI, AMP	3	0.946	9.623	51.03	11.904	0.002
15	Area, AMP. MDE	3	0.944	3.573	51.438	12.312	0.002
9	Area, EVI, MDE	3	0.939	3.204	52.374	13.248	0.001
18	EVI, AMT	2	0.719	10.858	58.593	19.467	<0.001
4	Area, EVI, AMT, AMP	4	0.975	23.354	58.602	19.476	<0.001
6	Area, EVI, AMT, MDE	4	0.97	29.533	60.236	21.11	<0.001
12	Area, AMT, AMP, MDE	4	0.969	18.676	60.547	21.421	<0.001
25	AMT	1	0.369	1	60.699	21.573	<0.001
17	EVI	1	0.239	1	62.568	23.442	<0.001
29	AMP	1	0.238	1	62.573	23.447	<0.001
26	AMT, AMP	2	0.571	8.641	62.834	23.708	<0.001
27	AMT, AMP, MDE	3	0.813	9.409	63.547	24.421	<0.001
28	AMT, MDE	2	0.539	1.566	63.555	24.429	<0.001
19	EVI, AMT, AMP	3	0.795	13.327	64.461	25.335	<0.001
1	Area	1	0.015	1	65.147	26.021	<0.001
31	MDE	1	0.014	1	65.156	26.03	<0.001
24	EVI, MDE	2	0.456	1.862	65.205	26.079	<0.001
8	Area, EVI, AMP, MDE	4	0.947	29.519	66.015	26.889	<0.001
21	EVI, AMT, MDE	3	0.741	20.79	66.77	27.644	<0.001
30	AMP, MDE	2	0.345	1.486	67.064	27.938	<0.001
23	EVI, AMP, MDE	3	0.721	15.651	67.545	28.419	<0.001
22	EVI, AMP	2	0.242	8.18	68.524	29.398	<0.001
16	Area, MDE	2	0.018	1.934	71.112	31.986	<0.001
20	EVI, AMT, AMP, MDE	4	0.813	39.687	78.523	39.397	<0.001
5	Area, EVI, AMT, AMP, MDE	5	0.985	45.524	83.337	44.211	<0.001

Supplementary Table S7. Summary for multiple regressions on larger-ranged rodent species richness (interpolated) along the northern slope of Mt. Taibai. N: number of variables; r^2: coefficient of determination; C.N.: condition number; AIC_c: model AIC_c score; D. A.: model AIC_c difference; W_i: model Akaike weight. Five explanatory variables: Area, EVI (enhanced vegetation index), AMT (annual mean temperature), AMP (annual mean precipitation) and MDE (mid-domain effect).

Model #	Variables	N	r^2	C.N.	AIC_c	D.A.	W_i
8	Area, EVI, AMP, MDE	4	0.994	8.856	28.138	0	0.46
12	Area, AMT, AMP, MDE	4	0.993	6.696	30.095	1.957	0.173
9	Area, EVI, MDE	3	0.979	7.816	30.296	2.158	0.157
6	Area, EVI, AMT, MDE	4	0.992	12.59	31.157	3.019	0.102
16	Area, MDE	2	0.954	1.999	31.869	3.73	0.071
30	AMP, MDE	2	0.941	1.148	34.603	6.465	0.018
15	Area, AMP, MDE	3	0.962	3.945	36.902	8.763	0.006
2	Area, EVI	2	0.922	4.414	37.632	9.493	0.004
13	Area, AMT, MDE	3	0.959	5.397	37.999	9.861	0.003
24	EVI, MDE	2	0.906	1.359	39.671	11.533	0.001
28	AMT, MDE	2	0.906	1.161	39.676	11.537	0.001
20	EVI, AMT, AMP, MDE	4	0.981	14.346	40.646	12.508	<0.001
23	EVI, AMP, MDE	3	0.942	3.119	41.701	13.563	<0.001
27	AMT, AMP, MDE	3	0.942	4.306	41.729	13.591	<0.001
31	MDE	1	0.815	1	41.9	13.762	<0.001
3	Area, EVI, AMT	3	0.928	11.602	44.031	15.893	<0.001
10	Area, AMT	2	0.857	2.967	44.296	16.158	<0.001
7	Area, EVI, AMP	3	0.923	5.409	44.762	16.623	<0.001
5	Area, EVI, AMT, AMP, MDE	5	0.994	39.172	46.384	18.245	<0.001
21	EVI, AMT, MDE	3	0.908	7.931	46.815	18.677	<0.001
1	Area	1	0.705	1	47.013	18.874	<0.001
11	Area, AMT, AMP	3	0.89	5.282	48.774	20.636	<0.001
14	Area, AMP	2	0.739	2.47	50.915	22.777	<0.001
4	Area, EVI, AMT, AMP	4	0.938	31.079	53.349	25.211	<0.001
17	EVI	1	0.31	1	56.359	28.221	<0.001
29	AMP	1	0.226	1	57.627	29.488	<0.001
25	AMT	1	0.187	1	58.168	30.029	<0.001
18	EVI, AMT	2	0.424	6.608	59.623	31.485	<0.001
19	EVI, AMT, AMP	3	0.693	11.372	60.022	31.884	<0.001
22	EVI, AMP	2	0.314	2.904	61.541	33.403	<0.001
26	AMT, AMP	2	0.226	4.256	62.861	34.723	<0.001

Supplementary Table S8. Summary for multiple regressions on smaller-ranged rodent species richness (interpolated) along the northern slope of Mt. Taibai. N: number of variables; r^2: coefficient of determination; C.N.: condition number; AIC_c: model AIC_c score; D. A.: model AIC_c difference; W_i: model Akaike weight. Five explanatory variables: Area, EVI (enhanced vegetation index), AMT (annual mean temperature), AMP (annual mean precipitation) and MDE (mid-domain effect).

Model #	Variables	N	r^2	C.N.	AIC_c	D.A.	W_i
29	AMP	1	0.838	1	22.036	0	0.293
25	AMT	1	0.838	1	22.091	0.055	0.285
26	AMT, AMP	2	0.884	4.256	23.6	1.563	0.134
18	EVI, AMT	2	0.875	6.608	24.418	2.381	0.089
22	EVI, AMP	2	0.862	2.904	25.511	3.475	0.052
30	AMP, MDE	2	0.851	1.057	26.386	4.349	0.033
10	Area, AMT	2	0.847	2.967	26.694	4.658	0.029
28	AMT, MDE	2	0.841	1.275	27.102	5.066	0.023
14	Area, AMP	2	0.839	2.47	27.251	5.215	0.022
11	Area, AMT, AMP	3	0.894	5.282	29.938	7.902	0.006
19	EVI, AMT, AMP	3	0.894	11.372	29.986	7.95	0.006
17	EVI	1	0.667	1	29.992	7.955	0.005
7	Area, EVI, AMP	3	0.89	5.409	30.384	8.348	0.005
21	EVI, AMT, MDE	3	0.889	9.755	30.468	8.432	0.004
27	AMT, AMP, MDE	3	0.885	4.758	30.882	8.846	0.004
3	Area, EVI, AMT	3	0.885	11.602	30.9	8.863	0.003
15	Area, AMP, MDE	3	0.874	5.445	31.867	9.83	0.002
23	EVI, AMP, MDE	3	0.863	3.954	32.819	10.783	0.001
24	EVI, MDE	2	0.712	1.592	33.618	11.581	<0.001
13	Area, AMT, MDE	3	0.847	4.918	33.99	11.953	<0.001
2	Area, EVI	2	0.691	4.414	34.419	12.382	<0.001
8	Area, EVI, AMP, MDE	4	0.938	7.348	35.146	13.11	<0.001
1	Area	1	0.45	1	35.518	13.482	<0.001
12	Area, AMT, AMP, MDE	4	0.929	6.504	36.511	14.474	<0.001
16	Area, MDE	2	0.568	2.123	38.104	16.068	<0.001
20	EVI, AMT, AMP, MDE	4	0.908	13.757	39.463	17.427	<0.001
4	Area, EVI, AMT, AMP	4	0.894	31.079	40.924	18.888	<0.001
9	Area, EVI, MDE	3	0.713	6.011	40.927	18.891	<0.001
6	Area, EVI, AMT, MDE	4	0.892	12.829	41.231	19.194	<0.001
31	MDE	1	0.026	1	41.197	19.761	<0.001
5	Area, EVI, AMT, AMP, MDE	5	0.941	39.6	52.839	30.802	<0.001

Supplementary Figure S9. Relationship between species turnover (represented as Sorensen dissimilarity index between paired elevational belts) and geographic distance. Results of linear regressions are presented.

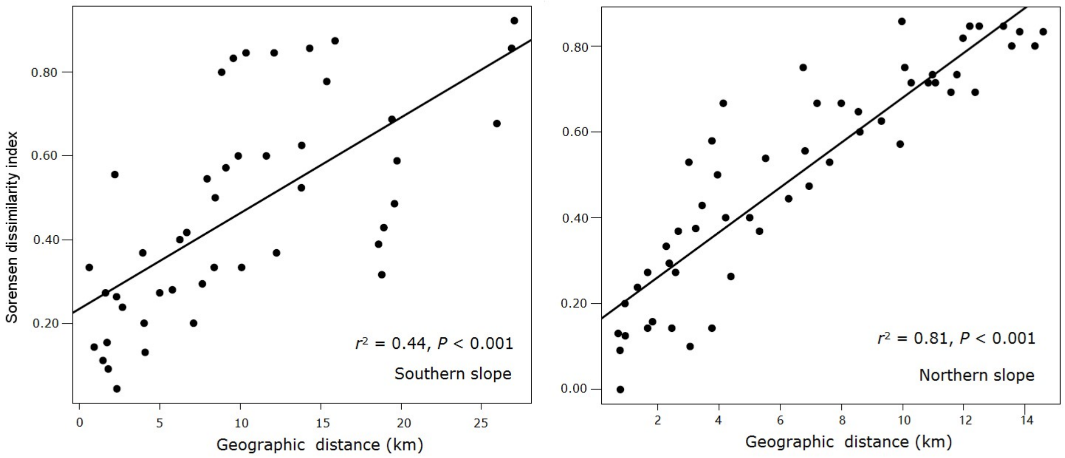

Supplementary Figure S10. Species accumulation curves for each elevational belt. Species added from literatures are excluded.

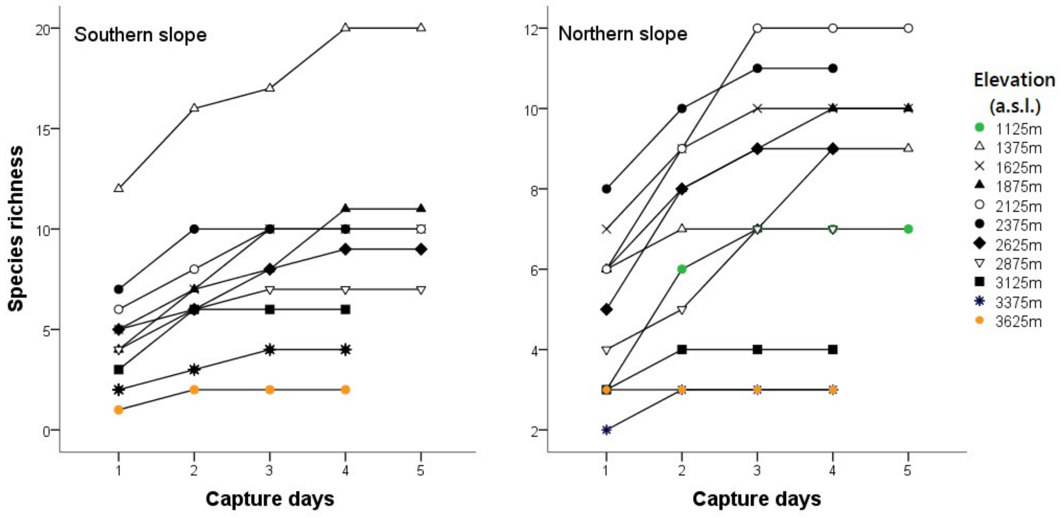

Supplement: Supplementary file 1 — Supplemental Information [file 41598_2017_9274_MOESM1_ESM.pdf]
